# HIT HAR: Human Image Threshing Machine for Human Activity Recognition Using Deep Learning Models

**DOI:** 10.1155/2022/1808990

**Published:** 2022-10-06

**Authors:** Alwin Poulose, Jung Hwan Kim, Dong Seog Han

**Affiliations:** ^1^Center for ICT and Automotive Convergence, Kyungpook National University, 80 Daehak-ro, Buk-gu, Daegu 41566, Republic of Korea; ^2^Graduate School of Electronic and Electrical Engineering, Kyungpook National University, 80 Daehak-ro, Buk-gu, Daegu 41566, Republic of Korea

## Abstract

In recent days, research in human activity recognition (HAR) has played a significant role in healthcare systems. The accurate activity classification results from the HAR enhance the performance of the healthcare system with broad applications. HAR results are useful in monitoring a person's health, and the system predicts abnormal activities based on user movements. The HAR system's abnormal activity predictions provide better healthcare monitoring and reduce users' health issues. The conventional HAR systems use wearable sensors, such as inertial measurement unit (IMU) and stretch sensors for activity recognition. These approaches show remarkable performances to the user's basic activities such as sitting, standing, and walking. However, when the user performs complex activities, such as running, jumping, and lying, the sensor-based HAR systems have a higher degree of misclassification results due to the reading errors from sensors. These sensor errors reduce the overall performance of the HAR system with the worst classification results. Similarly, radiofrequency or vision-based HAR systems are not free from classification errors when used in real time. In this paper, we address some of the existing challenges of HAR systems by proposing a human image threshing (HIT) machine-based HAR system that uses an image dataset from a smartphone camera for activity recognition. The HIT machine effectively uses a mask region-based convolutional neural network (R-CNN) for human body detection, a facial image threshing machine (FIT) for image cropping and resizing, and a deep learning model for activity classification. We demonstrated the effectiveness of our proposed HIT machine-based HAR system through extensive experiments and results. The proposed HIT machine achieved 98.53% accuracy when the ResNet architecture was used as its deep learning model.

## 1. Introduction

The human healthcare systems have a vital role in our daily life. Due to the busy lifestyle, these days, the lack of exercise causes serious health issues. Emerging technologies such as human activity recognition (HAR) systems [1] can monitor the users' activities in the healthcare system. Recent research trends in HAR show its wide variety of applications that include health and fitness monitoring [2], assisted living [3], context-enabled games and entertainment [4], social networking [5], and sports tracking [6]. In HAR, the system tracks the user's movements and classifies the user's activities based on the sensor reading. The existing HAR system includes vision-based [7], radiofrequency-based [8], or wearable sensor-based approaches [9]. The most common and low installation cost-based HAR technique is the wearable sensor-based approach. The sensor-based technique is location independent, and the user can easily hold the sensor during their activities. The sensor-based HAR approaches achieved a remarkable classification accuracy, and smartphone or smartwatch-based HAR is the most common system used for activity recognition. However, the sensor errors, sensor type, sensor position in the human body, and user's complex activities make the system more challenging for activity recognition. The HAR system has worst classification results when the user is in complex activity motion. On the other side, when the HAR system uses radio frequency (RF) signals for activity recognition, the system takes advantage of the wireless communication features to classify the user's activities. Compared with the sensor-based HAR approach, RF-based HAR is device-free, and the system does not need any physical sensing module. The device-free characteristics of radio frequency-based HAR provide reduction in energy consumption and privacy protection compared with the sensor or vision-based HAR systems. However, indoor channel conditions, non-line of sight conditions, and signal interference affect the performance of HAR, and the system faces difficulties in maintaining high accuracy levels. Besides these HAR approaches, the vision-based HAR system uses a camera that records the user's activities in a video sequence. The vision-based approach uses computer vision algorithms for activity recognition. Based on the camera type used in the HAR system, the video sequence from the vision approach is in the form of RGB videos [10], depth videos [11], or RGB-D videos [12]. Compared with sensor-based or radio frequency-based HAR approaches, the vision-based approach shows higher classification results for users' complex activities. However, user privacy, energy consumption, and deployment cost are the main challenges for the vision-based HAR approaches. In this paper, our research focuses on the vision-based HAR approach, and we propose a human image threshing (HIT) machine-based HAR system that addresses some of the existing vision-based HAR challenges. Our HIT machine-based HAR system uses a smartphone camera as an input device to record the users' activities. A mask region-based convolutional neural network (R-CNN) further processes the recorded activity videos for human body detection, a facial image threshing machine (FIT) for image cropping and resizing [13], and a deep learning model for activity recognition. Our HIT machine can generate HAR images from activity videos, human body detection from images, data cleaning and removal of irrelevant data, and activity classification using a deep learning model. We tested our HIT machine with different HAR experiments based on deep learning models, including visual geometry group (VGG) [14], Inception [15], ResNet [16], and EfficientNet [17] models. The results from the HIT machine show that the system always maintains the classification accuracy for activity recognition. We analyzed our HIT machine results with conventional HAR approaches that include inertial measurement unit (IMU) and stretch sensor-based approaches. The results show that the HIT machine outperforms the traditional sensor-based approaches with a higher level of accuracy for activity recognition. We also tested our pre-trained deep learning models with unseen HAR datasets and analyzed the classification performance. The key contributions from our HIT machine are stated as follows:

- We created a HAR dataset using a smartphone camera, IMU sensor, and stretch sensor. Our dataset consists of nine activities: sitting, standing, lying, walking, push up, dancing, sit-up, running, and jumping. It has 36, 558 image samples from smartphone cameras, 97,454 data samples from IMU sensors, and 7,850 data samples from stretch sensors. We used these datasets to validate our HIT machine, and the deep learning models can use our HAR datasets for training and testing without any computational complexity. We also collected HAR datasets for unseen datasets and tested them with pre-trained deep learning models.
- We proposed a HIT machine for activity recognition, and our HIT machine shows accurate classification results for basic (sitting, standing, and walking) and complex (running, jumping, and lying) activities. We tested our HIT machine with different deep learning models and analyzed the classification performance in terms of a confusion matrix, accuracy, loss, precision, recall, and F1 score. We also tested the pre-trained models with unseen HAR datasets and compared the performance of each model. We validated our HIT machine results with sensor-based HAR results and proved the impact of the HIT machine for activity recognition.

The rest of the paper is organized as follows: Section 2 discusses the existing HAR systems, recently proposed HAR systems with their advantages, and current HAR challenges for practical implementation.  Section 3 presents our proposed HIT machine-based HAR system, including mask R-CNN, FIT machine, and deep learning models.  Section 4 discusses our HAR experiments with the validation of our HIT machine in terms of the impact of various deep learning models, analysis of unseen datasets for pre-trained models, and the result comparison with conventional HAR approaches. Finally, Section 5 concludes our HIT machine-based HAR approach with future research directions.

## 2. Related Work

HAR has been studied for applications in healthcare monitoring, smart homes, security, medical imaging, robot/human interaction, personal assistants, and surveillance [18–20]. Many researchers have discussed various HAR approaches based on the technologies or algorithms used for activity recognition [21–25]. In this paper, our literature focuses on related work for HAR approaches that include sensors [26, 27], Wi-Fi [28], Wi-Fi, and sensors [29], vision [30, 31], and RFID [32]-based activity recognition. The HAR approaches from [26–32] provide significant performance improvements for HAR applications. However, the diversity of age, gender, and number of subjects, postural transitions, number of sensors and type of sensors, different body locations of wearable sensors or smartphones, missing values or labeling error, similar postures and datasets having complex activities, lack of ground truths, selection of appropriate datasets, and selection of sensors [33, 34] create challenges to the HAR implementation. This paper proposes a HIT machine-based HAR system to address some of these challenges with higher classification results.

The sensors-based HAR approaches are the most common and popular HAR systems. In sensor-based HAR, the system uses wearable sensors, smartphones, or smartwatches to collect data and identify the users' activity based on the sensor readings. Some of the recent HAR systems which take advantage of wearable sensors are discussed in [35–39]. These systems achieved a remarkable recognition accuracy in real time. However, mounting a wearable sensor in the human body is challenging, and the wearable sensor's position determines the system's performance. The wearable sensor-based HAR systems still need to optimize the location of sensors in the human body for complex activity. An alternative method for activity recognition is the smartphone-based HAR systems [40–43]. In smartphone-based HAR, the user holds the smartphone and performs the activities. Compared with wearable sensor-based approaches, the smartphone-based method is simple and easy to implement in any place without any external sensors. However, the position in which the smartphone is held and the modes such as texting and calling affect the system's performance. The smartphone or wearable sensors-based HAR approach still needs to improve the classification performance at a certain level, and current systems use deep learning models for activity recognition [44–47]. The deep learning HAR-based systems include convolutional neural network (CNN) [48], long short-term memory (LSTM) [49], LSTM-CNN [50], deep recurrent neural networks (DRNN) [51], generative adversarial networks (GAN) [52], extreme learning machine (ELM) [53], graph neural network (GNN) [54], and semi-supervised deep learning models [55, 56]. These systems use raw sensor reading or extract the signal features in the time/frequency domain for activity recognition. When the system uses the signal in the time domain, it extracts the variance, mean, maximum, minimum, and range values and uses these features as model inputs. On the other hand, If the signal is in the frequency domain, the system extracts the amplitude, skewness, kurtosis, and energy information as to its features and uses this input to the model. Compared with the raw input signal-based deep learning HAR approach, the feature-based approaches show better classification results [2]. However, the deep learning-based HAR approaches are not free from challenges. A large number of data samples for training, training time, the complexity of feature extraction, and human resources required for data collection are some of the main challenges of deep learning-based HAR approaches. These challenges reduce systems performance and require further classification improvements.

The RF-based HAR approaches use physical sensors, such as pressure, proximity, FM radio, microwave, or RFID for activity recognition [57–61]. In a radio frequency-based approach, the system takes advantage of the body attenuation and the channel fading characteristics for activity recognition. The basic principle of RF-based HAR systems is that the propagation of RF signals is affected by the human body movement, resulting in attenuation, refraction, diffraction, reflection, and multipath effects. These pattern differences in the received RF signals are the key ideas for activity recognition. Different activities lead to various patterns inside RF signals, and the system can use these features for classification. The RF-based systems consist of signal selection, model, signal processing, segmentation, feature extraction, and activity classification. In signal selection, the system uses Wi-Fi, ZigBee [63], RFID [64], frequency-modulated continuous-wave radar (FMCW) or acoustic devices. The system uses phase, frequency, amplitude, or raw signal for activity recognition depending on the signal selection. These factors determine the model of the HAR system. When the model is defined, the system uses signal processing techniques, including noise reduction, calibration, and redundant removal. After this, the system uses signal segmentation in the time or frequency domain. When segmentation performed, the time domain, frequency domain, time-frequency domain, or spatial domain features are extracted for classification. The deep learning models use extracted features for activity recognition. Compared with the wearable sensor-based HAR approach, the RF-based approach exploits the wireless communication features for activity recognition. These systems do not use any physical sensing module, thus reducing energy consumption and user privacy concern. Some of the RF-based HAR approaches are discussed in [65–68]. The RF-based systems discussed here have enhanced the HAR classification performance and opened many applications for detection, recognition, estimation, and tracking. However, the wireless channel conditions, signal interference, non-line-of-sight (NLOS) conditions, multi-user activity sensing, and limited sensing range make the systems more challenging. They require new theoretical models and open datasets for accurate classification.

The system uses a video sequence for activity monitoring when considering a vision-based HAR approach for activity recognition [69, 70]. The vision-based approach is best for multi-user activity recognition when privacy is not a significant concern. These systems use different computer vision algorithms on activity videos to predict the user's activities from videos or images. Some of the vision-based HAR approaches are proposed in [71–77]. These vision-based systems effectively use the video or image sequences and classify the users' activity by taking advantage of the recent deep learning models. Several review papers on the vision-based HAR systems are discussed in [78–80]. From vision-based HAR review discussions, the authors from [81] focus on the high level of visual processing, including human body modeling, understanding of human actions, and approaches to human action recognition. In [82], the authors presented the current state-of-the-art development of automated visual surveillance systems. They discussed the necessity of intelligent visual surveillance in commercial, law enforcement, and military applications. In [83], the paper reviews the advances in human motion capture and analysis from 2000 to 2006 and discusses the problems for future research to achieve automatic visual analysis of human movement. The review paper [84] analyzes the approaches taken to date within the computer vision, robotics, and artificial intelligence communities to represent, recognize, synthesize, and understand action. In [84], the authors pay more attention to identifying actions at different levels of complexity. Machine recognition of human activities is reviewed in [85], and the authors present a comprehensive survey of efforts to address the vision-based HAR systems. The paper [80] focuses on pedestrian detection, and [86] introduces a HAR system that recognizes the human behaviors from transit scenes. The most recent HAR systems are presented in [87–89]. These systems tried to improve the feature extraction techniques by introducing object detection, skeleton tracking, and human body poses. The vision-based HAR systems discussed here still have some challenges, such as processing high-quality videos or images, the complexity of the vision algorithms, the requirement for a higher graphics processing unit (GPU) processing power, the installation cost of the camera, and challenges from vision systems such as camera viewpoint, lighting, human body appearance, occlusion, and background clutter. These challenges make it more difficult for the vision-based approaches for real-time health monitoring.

So far, we have discussed different types of HAR approaches based on their technologies and algorithms used for activity recognition. In this paper, our research mainly focuses on the vision-based HAR approach, and we used our smartphones for data collection. We also collected data using IMU and stretch sensors, and the results from these sensors are compared with our proposed HIT machine. The experiment results show that the HIT machine is a practical HAR approach for healthcare applications and needs only a basic smartphone model for activity recognition.

## 3. Proposed HIT Machine-Based HAR System

**Figure 1:**
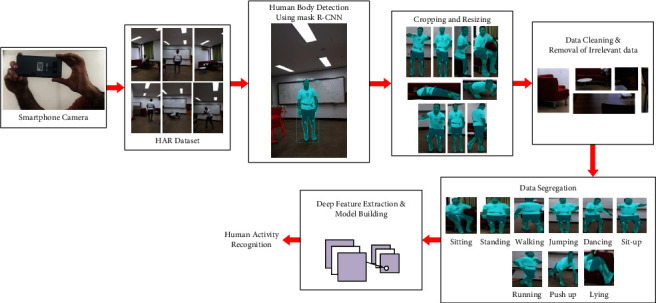
Proposed HIT machine-based HAR system.

The HIT machine consists of HAR dataset creation, data preprocessing, human body detection using mask R-CNN, image cropping and resizing, data cleaning and removal of irrelevant data, deep feature extraction, model building, and activity classification.  Figure 1 shows the framework of our proposed HIT machine-based HAR system.

**Figure 2:**
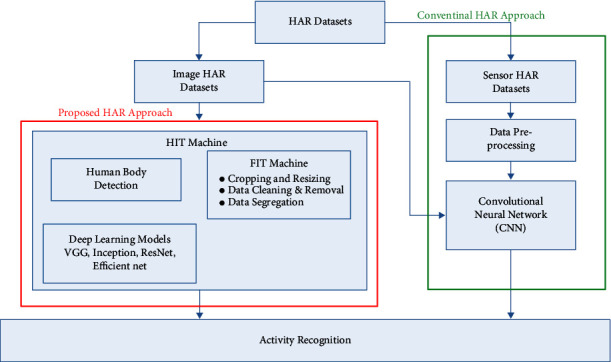
Flowchart of the proposed HIT machine.

We first started our data collection in the HIT machine by using android and iOS smartphones that record activity videos. Next, the HIT machine performs the data aggregation on the activity video sequences. The data aggregation gathers all activity data and presents it in a summarized format. Followed by the data aggregation process, our system uses a mask R-CNN algorithm for human body detection. After this, the HIT machine operates the FIT machine for image cropping and resizing when the human body is identified from images. The cropped and resized activity images are ready for the model to use for training and testing. Our HIT machine also used a data cleaning process that removes the unnecessary images from the HAR dataset. After the data cleaning process, the images are ready to be used for model training and testing. We extracted the features from the activity images and created a deep learning model that classifies user activities into nine groups. The output of the HIT machine is the classification results of user activities which include sitting, standing, lying, walking, push up, dancing, sit-up, running, and jumping. The flowchart of the proposed HIT machine is presented in Figure 2.

In the flowchart, the system starts with HAR datasets. The datasets include HAR images from smartphones, accelerometer and gyroscope readings from IMU sensors, and stretch sensor readings. The HAR image dataset is then divided into training, testing, and unseen datasets. We used our HIT machine in the image HAR dataset for human body detection and activity recognition. The HIT machine includes human body detection, data preprocessing using a FIT machine, and deep learning models for classification. A mask R-CNN-based object detection algorithm is used for human body detection. A FIT machine is used for data preprocessing, including image cropping, resizing, data cleaning, and data segregation. A deep learning model is used for the training, and the model classifies the user activities into different categories. The system uses deep learning models of VGG, Inception, ResNet, and EfficientNet. On the other hand, conventional HAR approaches use IMU and stretch sensor data for activity recognition with a CNN model. The CNN model also uses the HAR image dataset for activity recognition, and we compared the effect of our HIT machine (with and without HIT machine) for activity recognition. Further discussions of mask R-CNN, FIT machine operation, and the deep learning models are added in the following subsections.

### 3.1. Mask R-CNN

**Figure 3:**
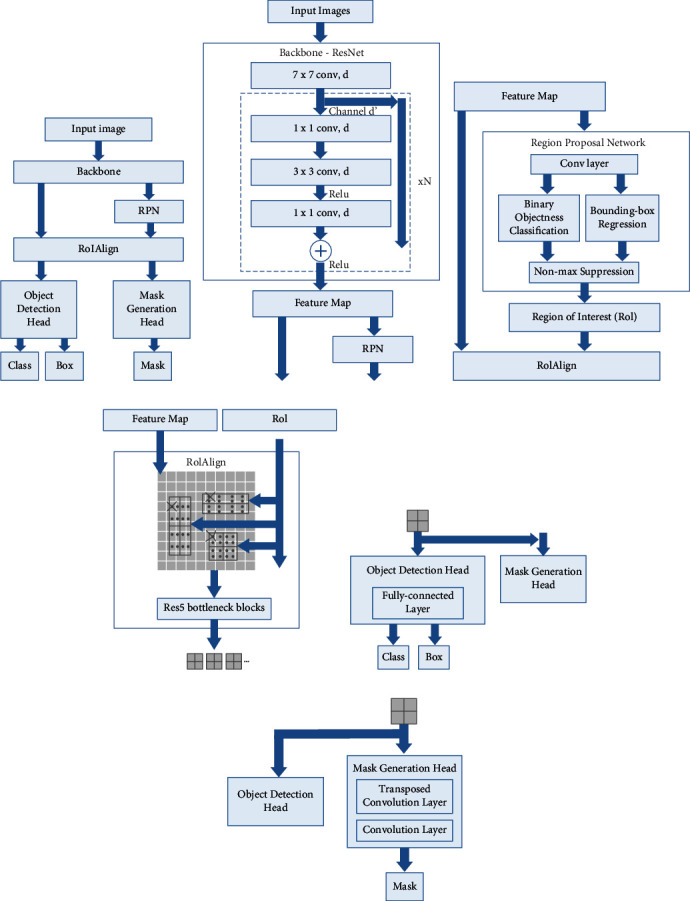
Mask R-CNN. (a) Structure. (b) Backbone. (c) RPN. (d) RoIAlign. (e) Object detection head. (f) Mask generation head.

In computer vision, mask R-CNN is widely used for object detection tasks [90]. The mask R-CNN separates different objects from a video or an image. The algorithm provides the object bounding boxes, classes, and mask information, and our HIT machine can effectively utilize this information for human body detection. The mask R-CNN from our HIT machine operates in two stages. First, the algorithm generates proposals about the regions where an object is located in the input image. Second, the algorithm predicts the object class and refines the bounding box. The algorithm also adds a mask in the pixel level of the object based on the first stage proposal. Compared with Fast/Faster R-CNN-based object detection approaches, the mask R-CNN-based approach has additional features such as a binary mask for each region of interest (RoI). Our system utilizes this binary mask feature for human body detection.  Figure 3 shows the structure of mask R-CNN.

**Figure 4:**
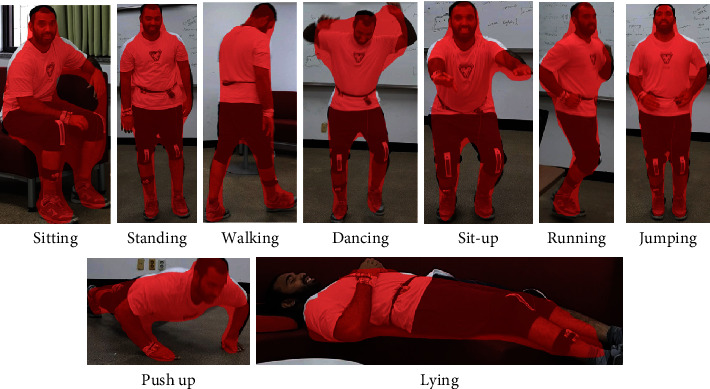
Human body detection using our HIT machine.

The mask R-CNN consists of a backbone, a region proposal network (RPN), a region of interest alignment layer (RoTAlign), an object detection head, and a mask generation head. The backbone of mask R-CNN is the primary feature extractor which uses residual networks (ResNets) with or without feature pyramid networks [91]. When our HAR images are fed into a ResNet backbone, the images go through multiple residual bottleneck blocks and turn into a feature map. The feature map contains the abstract information of input images, including different object instances, classes, and spatial properties. The feature map data are then fed into the RPN layer. In this layer, the network scans the feature map and RoI where the human body is located. The next step is to find each RoI from the feature map. This process is referred to as RoIAlign in Figure 3. The RoIAlign extracts the feature vectors from the feature map based on the RoI suggested by the RPN layer. The feature vectors are then converted into a fix-sized tensor for further processes. The outputs from RoIAlign are then processed by two parallel branches: object detection branch and mask generation branch. The object detection branch is a fully-connected layer that maps the feature vectors to the final classes and bounding box coordinates. The mask generation branch feeds the feature map into a transposed convolutional layer and convolutional layer. The output of mask generation branch is one binary segmentation mask that is generated for one class. Then the system picks the output mask based on the class prediction from the object detection branch.  Figure 4 shows the human body detection using our HIT machine for nine activities.

As shown in Figure 4, the mask R-CNN accurately detects the human body for nine activities without any detection error. The mask R-CNN used here is straightforward and has a small computational overhead that enables a fast system and rapid experimentation. For more details on mask R-CNN and its implementation, refer to [92–94].

### 3.2. FIT Machine

The HIT machine effectively uses our previously proposed FIT machine for image cropping and resizing [13]. The FIT machine is used to correct missing HAR datasets, remove irrelevant data, merge datasets on a massive scale, and crop and resize images. Our FIT machine converts input activity video sequences into the image output samples that consist of cropped, resized, and categorized activity images. The FIT machine contains a data receiver, a multi-task cascaded convolutional network (MTCNN), an image resizer [95], and a data segregator as the pre-trained Xception algorithm model [96]. The data receiver converts activity video sequences into images, and the MTCNN identifies the human faces from the activity images. The MTCNN used here consists of P-Net, R-Net, and O-Net layers. When the architecture detects the human faces, the input images enter the P-Net layer, which chooses the possible face frames from the input images. The R-Net layer in the MTCNN uses P-Net outputs as its inputs. The R-Net layer inspects the given initial frames from P-Net, then removes the face frames that do not reach a threshold score. Followed by the R-Net, the O-Net uses the output from the R-Net at the end. In the O-Net layer, it selects the best face frames from the given output from R-Net. Next, the images are passed through an image resizer, reducing the image size to 224×224 pixels. The last part of the FIT machine is a data segregator, which segregates the activity images into adequately labeled directories. The data segregator contains a pre-trained Xception model made by a depth-wise separable convolution layer. The depth-wise separable convolution layer used in the model splits each channel of the input and filter separately. The layer convolves them by each channel and later separates one element of 3 channels to be convoluted until all aspects have been convoluted. The architecture also has some shortcut structure that skips over the block of the depth-wise separable convolution layers. The model uses a categorical cross-entropy loss function as the metric loss measurement. For more details on the FIT machine, refer to [13].

### 3.3. Deep Learning Models

**Figure 5:**
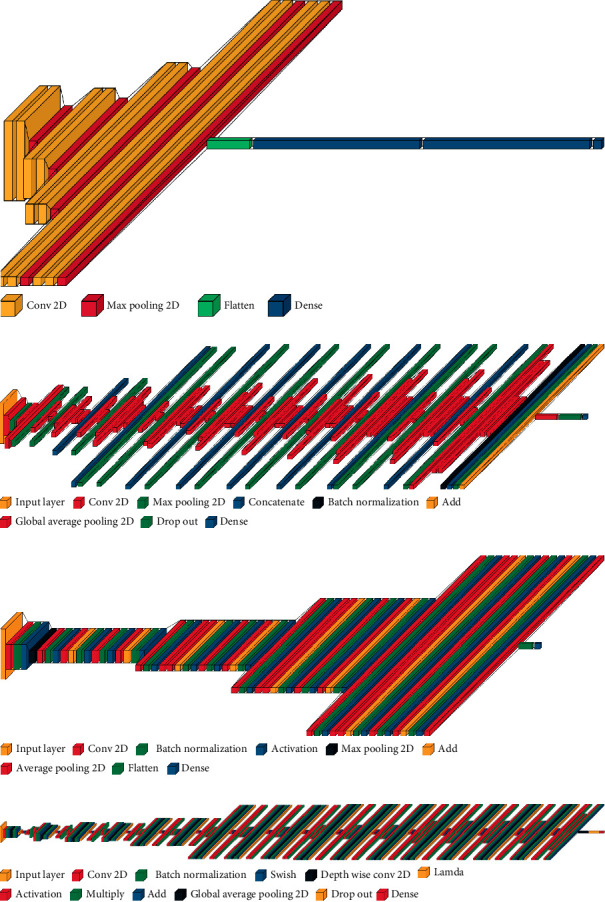
Deep learning models used in the HIT machine. (a) VGG. (b) Inception. (c) ResNet. (d) EfficientNet.

The last stage of the HIT machine is the deep learning models. Our HAR dataset is trained with deep learning models and classifies user activities into sitting, standing, lying, walking, push up, dancing, sitting, running, and jumping. The HAR dataset consists of image samples, and our system considers four image classification models VGG, ResNet, Inception, and EfficientNet, as the deep learning models.  Figure 5 shows the deep learning models used by our HIT machine.

The most common image classification model is the VGG model introduced by the visual graphics at University of Oxford [14]. The VGG model consists of 13 convolution layers, five pooling layers, and three dense layers. The VGG model is sequential in nature and uses many filters one after another. The architecture uses a stack of convolutional layers with different depths in different architectures followed by three fully-connected (FC) layers. The first two FC layers have 4,096 channels each, and the third FC performs the 1,000-way classification. The last layer is the soft-max layer that is used to normalize the classification vector. All the hidden layers in the VGG architecture use rectified linear unit (ReLU) as the activation function. The ReLU activation function is computationally efficient, and its results are in faster learning. The ReLU function also reduces the likelihood of vanishing gradient problems and improves the classification performance.  Figure 5(a) shows the architecture of the VGG network.

Next, our HIT machine used a deep learning model, which was developed by Google [16]. The GoogLeNet or Inception is a smaller network than the VGG model and uses an Inception module. The Inception module performs convolutions with different filter sizes on the input images, performs Max Pooling, and concatenates the result for the next Inception module. The architecture uses a 1×1 convolution operation which reduces the parameters drastically. This architecture is designed to solve the problem of computational expense, overfitting, and other deep learning model issues. The Inception model takes advantage of the multiple kernel filter sizes within the CNN, and rather than stacking them sequentially, it orders them to operate on the same level.  Figure 5(b) shows the Inception architecture used by our HIT machine. The architecture has nine inception modules stacked linearly and has 22 layers deep (27, including the pooling layers). It uses global average pooling at the end of the last inception module. Compared with VGG networks, Inception networks are more computationally efficient in terms of the number of parameters generated by the network and the computational cost incurred. For more details on the Inception model, refer to [16].

Our HIT machine also analyzed the impact of the ResNet architecture for activity recognition. The main idea of ResNet architecture is to avoid poor accuracy when the model uses deeper layers. This model is mainly designed for the gradient vanishing problem.  Figure 5(c) shows the ResNet architecture used by our HIT machine. The ResNet architecture is a 34-layer plain network inspired by VGG-19 networks, which adds shortcut connections. These shortcut connections then convert the ResNet architecture into the residual network. The first two layers of the model are the same as those of the Inception model. The model uses a 7×7 convolution layer with 64 output channels and a stride of 2 followed by the 3×3 maximum pooling layer. The major difference with ResNet is the batch normalization layer which is added after each convolutional layer. The inception model discussed previously uses four modules which are made up of Inception blocks. However, the ResNet architecture uses four modules which are made up of residual blocks. Each residual block uses several residual blocks with the same number of output channels. The first module from the architecture uses the number of channels that are the same as the input channel numbers. From the first residual block of each subsequent module, the number of channels is doubled compared with the previous module, and the height and width are halved. Compared with Inception architecture, the ResNet model is more straightforward, easy to modify, easy to optimize, and achieves higher accuracy when the depth of the network increases. For more details on ResNet architecture and its implementation, refer to [15].

At last, our HIT machine used a model called EfficientNet from Google for activity recognition [17]. In EfficientNet, a new scaling method called compound scaling is introduced. The model ResNet discussed before follows a conventional approach of scaling the dimensions arbitrarily and adding more layers. However, if the model scales the dimensions by a fixed amount simultaneously and does so uniformly, the model achieves better performance. The user can decide the scaling coefficients. EfficientNet architecture is a convolutional neural network architecture with different scaling methods. In EfficientNet, the architecture uniformly scales all depth/width/resolution dimensions using a compound coefficient. Compared with conventional ways that arbitrarily scale these factors, the scaling method in the EfficientNet architecture uniformly scales network width, depth, and resolution with a set of fixed-scaling coefficients.  Figure 5(d) shows the EffientNet architecture used by our HIT machine. The main building block of this architecture consists of mobile inverted bottleneck Convolution (MBConv), to which squeeze-and-excitation optimization is added. The MBConv layer is similar to the inverted residual blocks used in MobileNet v2 [97]. The MBConv creates a shortcut connection between the beginning and end of a convolutional block. The input activation maps are first expanded using 1×1 convolutions, increasing the depth of the feature maps. 3×3 depth-wise convolutions and point-wise convolutions follow this, and this structure reduces the number of channels in the output feature map. The shortcut connections connect the narrow layers, while the wider layers are present between the skip connections. This form of structure decreases the overall number of operations required as well as the model size. For more details on the EfficientNet architecture and its implementation, refer to [17].

## 4. Experiment Results and Analysis

We collected HAR datasets from different users to validate our proposed HIT machine-based HAR approach. There were 10 volunteers for data collection, consisting of five members for the training dataset and five for the unseen dataset. The demographic information of participants is given in Table 1.

**Figure 6:**
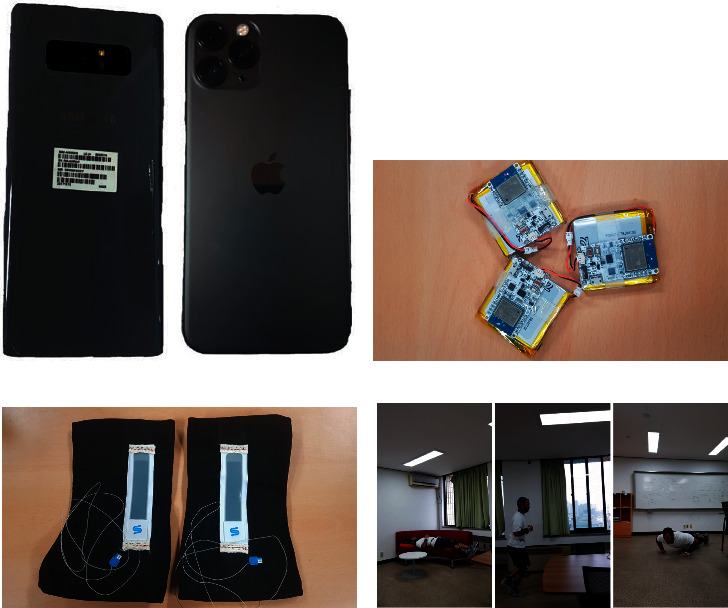
Experiment setup. (a) Smartphones. (b) IMU sensor. (c) Stretch sensor. (d) Experiment area.

We used Samsung galaxy note eight and iPhone 11 pro smartphone models for video recording. The smartphones were kept stationary during the initial stage of the experiment and moved their positions based on the user's motions. The users made their activities within the 15 m experiment area. We also used the IMU and stretch sensors and recorded the sensor reading from the users' activities during the experiment time. Our conventional HAR approaches use the sensor reading for activity recognition, and we compared these HAR results with our HIT machine approach.  Figure 6 shows the smartphones, IMU and stretch sensors, and experiment area involved in the HAR data collection.  Table 2 summarizes our system configurations and hyperparameters used for model training and testing.

**Figure 7:**
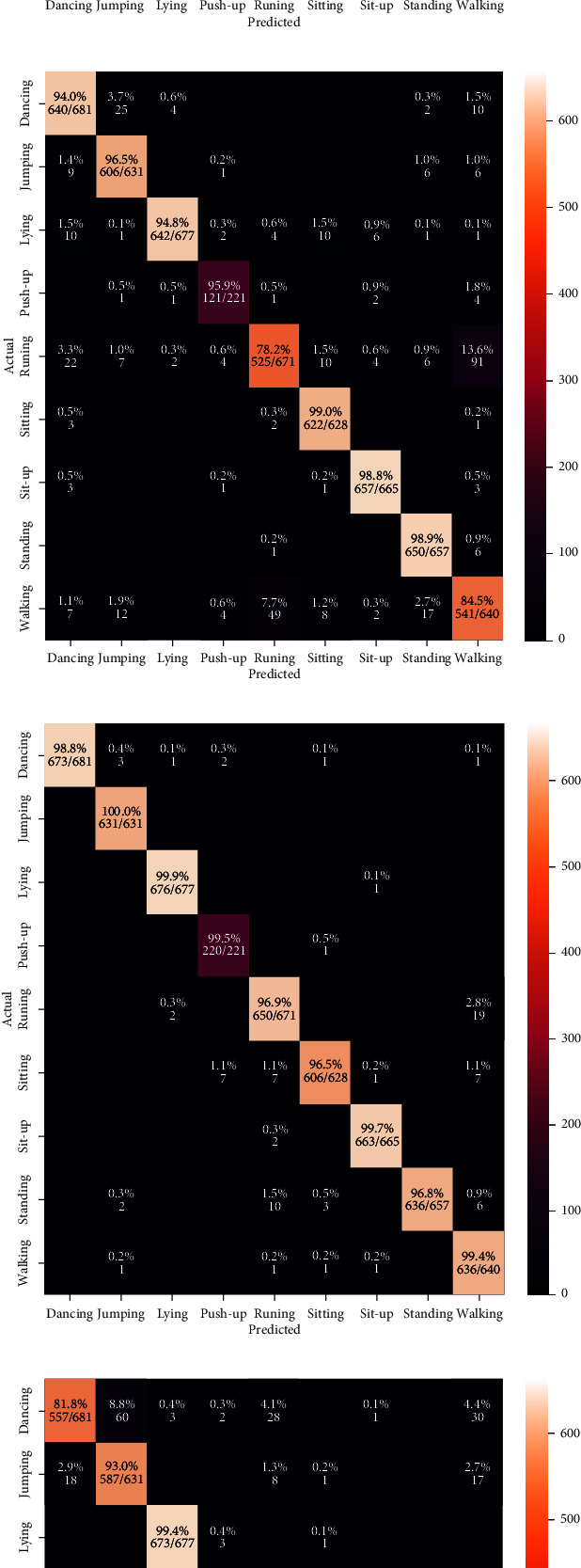
The confusion matrix results. (a) VGG. (b) Inception. (c) ResNet. (d) EfficientNet.

**Figure 8:**
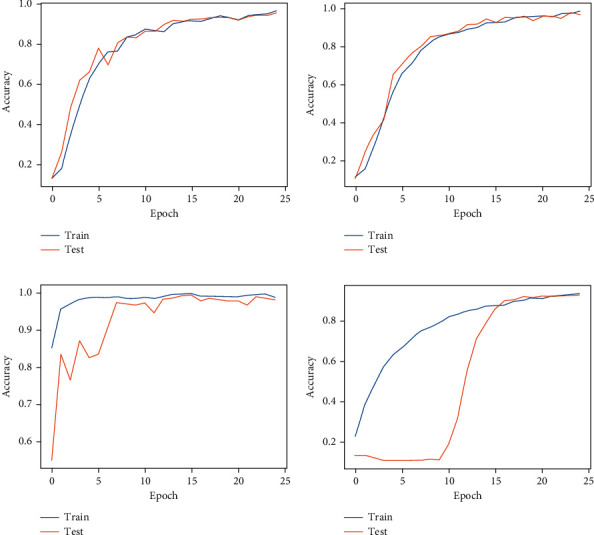
Deep learning models accuracy plots. (a) VGG. (b) Inception. (c) ResNet. (d) EfficientNet.

**Figure 9:**
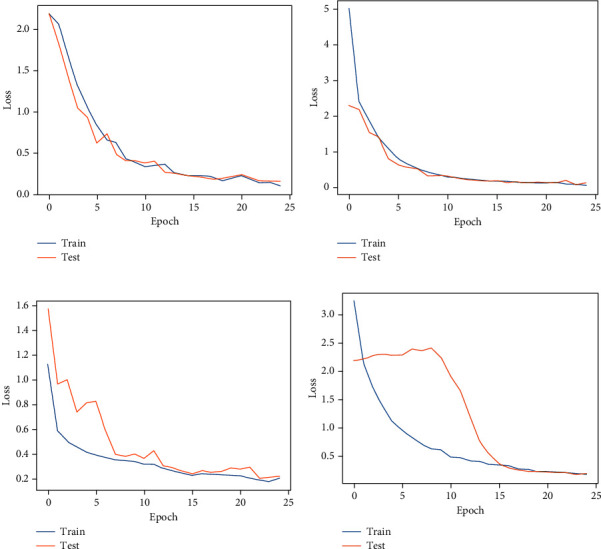
Deep learning models loss plots. (a) VGG. (b) Inception. (c) ResNet. (d) EfficientNet.

We started the analysis of the HIT machine by implementing deep learning models, such as VGG, Inception, ResNet, and EfficientNet. We tested these models with our HAR dataset, and Figure 7 shows the classification results from each model. We used confusion matrices to analyze each model, summarizing the classification performance. The color bars indicate the number of samples populated in a specific area. When the data samples are higher, the color becomes lighter and vice versa. The results observed in confusion matrices show that the ResNet architecture has the highest classification performance compared with other models and achieved a 98.53% model accuracy, 0.20 model loss, 98.56% precision, 98.53% recall, and 98.54% F1 scores. The VGG model reached 96.38% model accuracy with 0.09 model loss, 96.58% precision, 96.38% recall, and 96.36% F1 score as shown in Figure 7(a). The VGG model has a higher classification accuracy for sitting, sit-up, standing, and walking activities. The model has the highest misclassification error for running. Some of the running activity is misclassified as walking.  Figure 7(b) shows the classification results from the Inception model. This model achieved a 93.18% classification accuracy with 0.13 model loss, 93.18% precision and recall, and 93.11% F1 scores, which are worse performances than the results obtained by the VGG model. Furthermore, Figure 7(c) shows the best classification results from our HIT machine based on ResNet architecture. The ResNet architecture showed the best model accuracy with the least classification errors. However, the model loss is higher than other models and needs higher computation time than VGG and Inception models. This model maintains the classification accuracy for basic and complex activities, and the model is the best choice for HIT machine-based activity recognition.  Figure 7(d) shows our last deep learning model results from EfficientNet. The EfficientNet reached 89.94% for classification accuracy with 0.21 model loss, 90.19% precision, and 89.94% recall and F1 score, which has worse HAR performance than VGG, Inception, and ResNet models. The higher level of classification error from EfficientNet shows that this model is unsuitable for our HIT machine-based activity recognition. Figures 8 and 9 show the deep learning models accuracy and loss plots, and Table 3 summarizes their performance.

In Table 3, we used the accuracy, loss, precision, recall, and F1 score parameters for performance evaluation. The following equations from [98] define these parameters.

(1) $$\begin{array}{c} Accuracy=\frac{TP+TN}{TP+TN+FP+FN}\text{,}\\ Loss=-\overset{outputsize}{\underset{i-1}{\sum }}y_{i}.\;\log\hat{y_{i}}\text{,}\\ Precision=\frac{TP}{TP+FP}\text{,}\\ Recall=\frac{TP}{TP+FN}\text{,}\\ F1=\frac{2\times Recall\times Precision}{Recall+Precision}\text{,} \end{array}$$

where the variables TP, TN, FP, and FN are defined as true positive, true negative, false-positive and false-negative in a given experiment. In the loss function, *y*_*i*_ is the *ith* scalar value in the model output, $\hat{y_{i}}$ is the corresponding target value, and the output size is the number of scalar values in the model output. From the results in Table 3, the ResNet architecture outperforms the other deep learning models with an average value of 98.53%. These results indicate that the system trained with the ResNet model is the best choice for activity recognition.

When we consider the training time results from Table 3, it shows that the ResNet-based HAR approach has a higher training time (600 s) than other models. This is due to the deep architecture of ResNet, and the system takes more time to train the model. However, the activity recognition results from ResNet compensate for the training time when considering the overall system performance (6.44% of classification improvements than EfficientNet model-based HAR system). In the case of VGG model-based HAR, the system achieved the most down training time (240 s) compared with other models and reached good classification results for activity recognition. The Inception model-based HAR system has a 60 s time difference for model training compared with the EfficientNet model. The EfficientNet has a lower training time (300 s) than the Inception model-based (360 s) HAR system. However, the EfficientNet-based HAR approach shows worst classification results than other models.

To further validate our HIT machine performance, we tested the pre-trained deep learning models with unseen HAR datasets. We collected another set of HAR datasets and tested them with our pre-trained models.  Table 4 summarizes the results for unseen datasets from pre-trained models. The results in Table 4 show that the ResNet architecture achieved 72.13% for classification accuracy with 72.25% precision, 72.92% recall, and 72.95% F1 scores. These results outperformance the other pre-trained models. However, the computational complexity of this architecture makes it more practically challenging for real-time HAR applications. The classification accuracy from the Inception model shows that the model reached 65.17% for classification accuracy with 65.21% precision, 65.08% recall, and 65.59% F1 scores. The results from the Inception-based pre-trained model give better results than VGG and EfficientNet pre-trained models. In the case of VGG based pre-trained model, the system shows 61.85% for classification accuracy with 61.73% precision, 61.48% recall, and 61.47% F1 scores. The EfficientNet pre-trained model-based HAR system shows 57.42% for classification accuracy with 57.48% precision, 57.43% recall, and 57.72% F1 scores. These results show the worst classification results compared with other pre-trained models, and the approach is unsuitable for image-based HAR systems.

**Figure 10:**
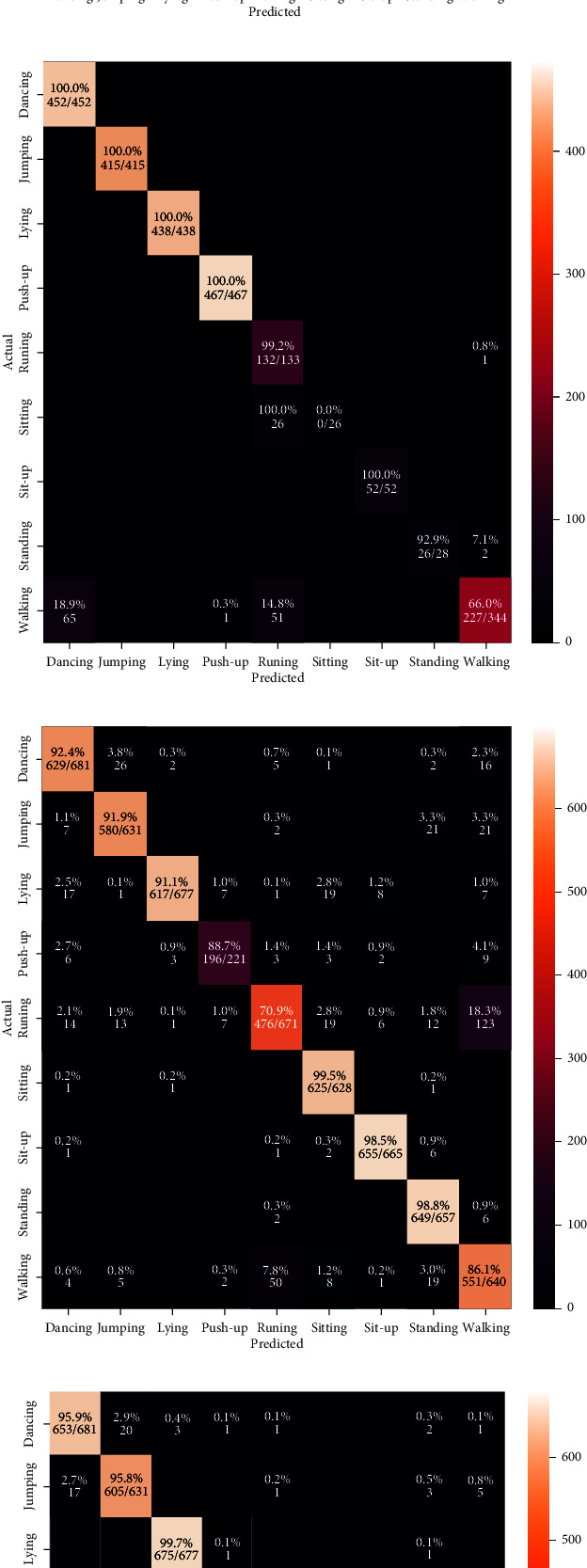
The confusion matrix results. (a) IMU sensor-based approach. (b) Stretch sensor-based approach. (c) HAR without HIT machine. (d) Proposed HIT machine-based approach.

Next, we validated our HIT machine results with sensor-based HAR approaches and image-based HAR without HIT machine.  Figure 10 shows the classification results from our HIT machine, HAR without HIT machine, and sensor-based techniques. This analysis uses a 2D CNN model for activity recognition. The CNN model is computationally lighter than other deep learning models and easily fits IMU and stretch sensor datasets.  Figure 10(a) shows the classification results from IMU sensor-based HAR approach. The results show that the IMU sensor approach reached 90.71% of classification accuracy with 0.27 model loss, 90.47% precision, 90.71% recall, and 90.00% F1 scores. The activities that include running, sitting, sit-up, standing, and walking have higher classification errors due to the similarities of IMU sensor data. The model fails to classify these activities, increasing the classification errors in the HAR system. When the system uses a stretch sensor instead of an IMU sensor, the classification performance has a 3% improvement. The stretch sensor-based HAR system achieved 93.80% of classification accuracy with 0.27 model loss, 94.16% precision, 93.80% recall, and 93.20% F1 scores.  Figure 10(b) shows the classification results from stretch sensor-based HAR approach. The stretch sensor data are more stable than the IMU sensor and have accurate HAR results. The activities that include sitting and walking have higher classification errors than the IMU sensor-based approach. The stretch sensor-based HAR approach is reasonable if the system cost is not a primary concern. The prohibitive cost of the stretch sensor makes the system more challenging for practical health care applications. Next, we analyzed a HAR approach that uses image data without a HIT machine.  Figure 10(c) shows the results from a HAR without HIT machine. The HAR system without HIT machine reached 90.98% of classification accuracy with 0.20 model loss, 91.24% precision, 90.98% recall, and 90.90% F1 scores. These results indicate the significance of the HIT machine. Compared with the results from Figure 10(d), the system without a HIT machine has a higher classification error and shows the worst performance for both basic and complex activities. The results from Figure 10(d) show the classification performance of the HIT machine, which has the best performance compared with other HAR approaches. The system achieved a 6.01% accuracy improvement compared with the IMU sensor-based approach and 2.4% accuracy improvement compared with the stretch sensor-based approach. The system also has a 5.3% accuracy improvement compared with the HAR approach without HIT machine. Our proposed HIT machine-based HAR system show 96.28% of classification accuracy with 0.09 model loss, 96.26% precision, 96.28% recall, and 96.27% F1 scores.  Table 5 summarizes the performance of each approach in terms of accuracy, loss, precision, recall, and F1 score. From Table 5 results, the HIT machine shows the highest classification results than the sensor-based and without HIT machine-based HAR approaches. The results indicate the impact of the HIT machine-based activity recognition for complex activities.

The training time results from Table 5 indicate that the stretch sensor-based HAR system shows the best training time (120 s) than the other HAR systems. This is due to the small number of data samples from the stretch sensor dataset. In the case of the IMU sensor-based HAR approach, the system has a 300 s training time, which is 180 s higher than the stretch sensor-based HAR approach. Also, the classification accuracy from the IMU sensor-based HAR approach is 3.09% lower than the stretch sensor-based approach. The proposed HIT machine-based HAR approach shows 340 s training time, which is lower than HAR without HIT machine-based approach (480 s). The training time results from our proposed HIT machine indicate that the approach reduced 140 s of training time compared with HAR without a HIT machine-based approach.

From the experiment and result analysis, it can be seen that the HIT machine-based HAR approach has a significant role in activity recognition. The proposed HAR system addresses the primary vision-based HAR system's challenge, such as processing high-quality images. We used image cropping, resizing, and data cleaning to make the system can perform the high-quality images without compromising the classification results. Our system takes advantage of the mask R-CNN algorithm, which is computationally lighter than other vision algorithms. The proposed method also solves the camera viewpoint and background clutter issues by considering the smartphone camera's wide-angle feature. The classification results from the HIT machine show that the proposed HAR approach is a valid method for healthcare applications, including abnormal activity detection, elderly care in homes, and disabled assistance. The extended versions of HIT machines are helpful in other applications, including intelligent environments, indoor navigation [99], security and surveillance, and people monitoring [100].

## 5. Conclusion

This paper proposed a HIT machine-based HAR system for healthcare applications. The proposed HIT machine approach effectively utilizes the advantages of the mask R-CNN for human body estimation and enhances the performance of the HAR. The classification results from our experiments indicate that the proposed HIT machine has better classification results than conventional sensor-based HAR approaches. The traditional sensor-based HAR systems are not free from sensor errors, showing very poor classification results for complex activities. The proposed HIT-based HAR system is suitable for basic and complex user movements and maintains its classification accuracy in all user motions. Our HAR classification results and analysis show the influence of the HIT machine for activity recognition. The proposed HIT machine-based HAR system is a suitable healthcare option if HAR systems use a camera as their input device. We validated our proposed HIT machine-based HAR system for human activity recognition through extensive experiments and analysis. To improve the classification performance, we intend to use a sensor fusion technique that combines the image and sensor data for activity recognition in our future work. Furthermore, we will consider the most popular public datasets (UCI- human activity recognition using smartphones dataset) for future research and compare our HAR datasets' performance with public datasets.

## Figures and Tables

**Table 1:** Demographics of participants.

Subjects	1	2	3	4	5	6	7	8	9	10
Age	30	21	35	22	28	26	30	32	35	23
Height (cm)	175	180	172	160	174	162	176	165	168	159
Weight (kg)	80	84	87	60	70	58	78	62	85	57
Gender	M	M	M	F	M	F	M	F	M	F
Training dataset	√	√		√			√			√
Unseen dataset			√		√	√		√	√	

**Table 2:** System configurations and hyperparameters used for model training and testing.

System configuration	Description
Processor	Intel® core^TM^ i7-11700k
RAM	32 GB
Graphics card	GeForce RTX^TM^ 3070 Ti
Python version	3.8
Tensorflow version	tf-nightly = = 2.6.0
Keras version	2.6.0
cuDNN library	cuDNN v8.1.0
CUDA version	CUDA toolkit 11.2.0
Model parameter	Value
Ratio of training data to overall data	0.70
Input image size	224 × 224
Number of channels	1
Optimizer	Adam
Learning rate	0.02
Batch size	128
Loss	Categorical cross-entropy
Number of classes	9
Epochs	25

**Table 3:** Performance comparison of deep learning models used in the HIT machine.

Deep learning model	Accuracy	Loss	Precision	Recall	F1 score	Training time (secs)
VGG	96.38	0.09	96.58	96.38	96.36	240
Inception	93.18	0.13	93.18	93.18	93.11	360
ResNet	98.53	0.20	98.56	98.53	98.54	600
EfficientNet	89.94	0.21	90.19	89.94	89.94	300

**Table 4:** Performance comparison of pre-trained deep learning models for unseen HAR datasets.

Pre-trained model	Accuracy	Precision	Recall	F1 score
VGG	61.85	61.73	61.48	61.47
Inception	65.17	65.21	65.08	65.59
ResNet	72.13	72.25	72.92	72.95
EfficientNet	57.42	57.48	57.43	57.72

**Table 5:** Performance comparison of HAR approaches.

HAR approach	Accuracy	Loss	Precision	Recall	F1 score	Training time (secs)
IMU sensor	90.71	0.27	90.47	90.71	90.00	300
Stretch sensor	93.80	0.27	94.16	93.80	93.20	120
HAR without HIT machine	90.98	0.20	91.24	90.98	90.90	480
Proposed HIT machine	96.28	0.09	96.26	96.28	96.27	340

## Data Availability

The data used to support the findings of this study have not been made available because of the privacy of the research participant.

## References

[1] Ronald M., Poulose A., Han D. S. (2021). iSPLInception: an inception-ResNet deep learning architecture for human activity recognition. *IEEE Access*.

[2] Steven Eyobu O., Han D. S. (2018). Feature representation and data augmentation for human activity classification based on wearable IMU sensor data using a deep LSTM neural network. *Sensors*.

[3] van Kasteren T. L. M., Englebienne G., Kröse B. J. A. (2010). An activity monitoring system for elderly care using generative and discriminative models. *Personal and Ubiquitous Computing*.

[4] Thu N. T. H., Han D. S., HAR H. (2021). A hierarchical hybrid deep learning architecture for wearable sensor-based human activity recognition. *IEEE Access*.

[5] Mehran R., Oyama A., Shah M. Abnormal crowd behavior detection using social force model.

[6] Ohgi Y., Yasumura M., Ichikawa H., Miyaji C. (2000). *Analysis of Stroke Technique Using Acceleration Sensor IC in Freestyle Swimming*.

[7] Ullah H. A., Letchmunan S., Zia M. S., Butt U. M., Hassan F. H. (2021). Analysis of Deep Neural Networks for Human Activity Recognition in Videos–A Systematic Literature Review. *IEEE Access*.

[8] Liu J., Teng G., Hong F. (2020). Human activity sensing with wireless signals: a survey. *Sensors*.

[9] Ashry S., Ogawa T., Gomaa W. (2020). Charm-deep: continuous human activity recognition model based on deep neural network using IMU sensors of smartwatch. *IEEE Sensors Journal*.

[10] Ren X., Gu C. Figure-ground Segmentation Improves Handled Object Recognition in Egocentric Video.

[11] Oikonomidis I., Kyriazis N., Argyros A. A. (2011). Efficient Model-Based 3D Tracking of Hand Articulations Using Kinect. *BmVC*.

[12] Lei J., Ren X., Fox D. Fine-grained kitchen activity recognition using rgb-d.

[13] Kim J. H., Poulose A., Han D. S. (2021). The extensive usage of the facial image threshing machine for facial emotion recognition performance. *Sensors*.

[14] Simonyan K., Zisserman A. (2014). Very Deep Convolutional Networks for Large-Scale Image Recognition. https://arxiv.org/abs/1409.1556.

[15] He K., Zhang X., Ren S., Sun J. Deep residual learning for image recognition.

[16] Szegedy C., Vanhoucke V., Ioffe S., Shlens J., Wojna Z. Rethinking the inception architecture for computer vision.

[17] Tan M., Le Q. Efficientnet: rethinking model scaling for convolutional neural networks.

[18] Rodriguez-Borbon J. M., Ma X., Roy-Chowdhury A. K., Najjar W. A. (2020). Heterogeneous acceleration of HAR applications. *IEEE Transactions on Circuits and Systems for Video Technology*.

[19] Ranasinghe S., Al Machot F., Mayr H. C. (2016). A review on applications of activity recognition systems with regard to performance and evaluation. *International Journal of Distributed Sensor Networks*.

[20] Yuan G., Wang Z., Meng F., Yan Q., Xia S. (2019). An Overview of Human Activity Recognition Based on Smartphone. *Sensor Review*.

[21] Jobanputra C., Bavishi J., Doshi N. (2019). Human activity recognition: a survey. *Procedia Computer Science*.

[22] Chen Y., Shen C. (2017). Performance analysis of smartphone-sensor behavior for human activity recognition. *IEEE Access*.

[23] Vrigkas M., Nikou C., Kakadiaris I. A. (2015). A review of human activity recognition methods. *Frontiers in Robotics and AI*.

[24] Fu B., Damer N., Kirchbuchner F., Kuijper A. (2020). Sensing technology for human activity recognition: a comprehensive survey. *IEEE Access*.

[25] Ramanujam E., Perumal T., Padmavathi S. (2021). Human activity recognition with smartphone and wearable sensors using deep learning techniques: a review. *IEEE Sensors Journal*.

[26] De-La-Hoz-Franco E., Ariza-Colpas P., Quero J. M., Espinilla M. (2018). Sensor-based datasets for human activity recognition–a systematic review of literature. *IEEE Access*.

[27] Lara O. D., Labrador M. A. (2013). A survey on human activity recognition using wearable sensors. *IEEE communications surveys & tutorials*.

[28] Alazrai R., Hababeh M., Alsaify B. A., Ali M. Z., Daoud M. I. (2020). An end-to-end deep learning framework for recognizing human-to-human interactions using Wi-Fi signals. *IEEE Access*.

[29] Muaaz M., Chelli A., Abdelgawwad A. A., Mallofré A. C., Pätzold M. (2020). WiWeHAR: multimodal human activity recognition using wi-fi and wearable sensing modalities. *IEEE Access*.

[30] Popoola O. P., Wang K. (2012). Video-based abnormal human behavior recognition—a review. *IEEE Transactions on Systems, Man, and Cybernetics, Part C (Applications and Reviews)*.

[31] Beddiar D. R., Nini B., Sabokrou M., Hadid A. (2020). Vision-based human activity recognition: a survey. *Multimedia Tools and Applications*.

[32] Wang F., Liu J., Gong W. (2021). Multi-adversarial in-car activity recognition using RFIDs. *IEEE Transactions on Mobile Computing*.

[33] Ahad M. A. R., Antar A. D., Ahmed M. (2020). IoT Sensor-Based Activity Recognition. *IoT Sensor-Based Activity Recognition*.

[34] Antar A. D., Ahmed M., Ahad M. A. R. Challenges in sensor-based human activity recognition and a comparative analysis of benchmark datasets: a review.

[35] Saengthong P., Laitrakun S. Fusion approaches of heterogeneous multichannel CNN and LSTM models for human activity recognition using wearable sensors.

[36] Lu J., Zheng X., Sheng M., Jin J., Yu S. (2020). Efficient human activity recognition using a single wearable sensor. *IEEE Internet of Things Journal*.

[37] Yen C.-T., Liao J.-X., Huang Y.-K. (2020). Human daily activity recognition performed using wearable inertial sensors combined with deep learning algorithms. *IEEE Access*.

[38] Lawal I. A., Bano S. (2020). Deep human activity recognition with localisation of wearable sensors. *IEEE Access*.

[39] Tang Y., Teng Q., Zhang L., Min F., He J. (2021). Layer-wise training convolutional neural networks with smaller filters for human activity recognition using wearable sensors. *IEEE Sensors Journal*.

[40] Qi W., Su H., Aliverti A. (2020). A smartphone-based adaptive recognition and real-time monitoring system for human activities. *IEEE Transactions on Human-Machine Systems*.

[41] Wang A., Chen G., Yang J., Zhao S., Chang C.-Y. (2016). A comparative study on human activity recognition using inertial sensors in a smartphone. *IEEE Sensors Journal*.

[42] Wang A., Zhao S., Zheng C., Chen H., Liu L., Chen G. (2021). HierHAR: sensor-based data-driven hierarchical human activity recognition. *IEEE Sensors Journal*.

[43] Chen Z., Jiang C., Xiang S., Ding J., Wu M., Li X. (2020). Smartphone sensor-based human activity recognition using feature fusion and maximum full a posteriori. *IEEE Transactions on Instrumentation and Measurement*.

[44] Abbaspour S., Fotouhi F., Sedaghatbaf A., Fotouhi H., Vahabi M., Linden M. (2020). A comparative analysis of hybrid deep learning models for human activity recognition. *Sensors*.

[45] Li X., Wang Y., Zhang B., Ma J. (2020). PSDRNN: an efficient and effective HAR scheme based on feature extraction and deep learning. *IEEE Transactions on Industrial Informatics*.

[46] Xu C., Chai D., He J., Zhang X., Duan S. (2019). InnoHAR: a deep neural network for complex human activity recognition. *IEEE Access*.

[47] Ihianle I. K., Nwajana A. O., Ebenuwa S. H., Otuka R. I., Owa K., Orisatoki M. O. (2020). A deep learning approach for human activities recognition from multimodal sensing devices. *IEEE Access*.

[48] Kim E. (2020). Interpretable and accurate convolutional neural networks for human activity recognition. *IEEE Transactions on Industrial Informatics*.

[49] Barut O., Zhou L., Luo Y. (2020). Multitask LSTM model for human activity recognition and intensity estimation using wearable sensor data. *IEEE Internet of Things Journal*.

[50] Xia K., Huang J., Wang H. (2020). LSTM-CNN architecture for human activity recognition. *IEEE Access*.

[51] Munoz-Organero M. (2019). Outlier detection in wearable sensor data for human activity recognition (HAR) based on DRNNs. *IEEE Access*.

[52] Li X. a., Luo J., Younes R. ActivityGAN: generative adversarial networks for data augmentation in sensor-based human activity recognition.

[53] Chen Z., Jiang C., Xie L. (2019). A novel ensemble ELM for human activity recognition using smartphone sensors. *IEEE Transactions on Industrial Informatics*.

[54] Mondal R., Mukherjee D., Singh P. K., Bhateja V., Sarkar R. (2021). A new framework for smartphone sensor-based human activity recognition using graph neural network. *IEEE Sensors Journal*.

[55] Zhu Q., Chen Z., Soh Y. C. (2019). A novel semisupervised deep learning method for human activity recognition. *IEEE Transactions on Industrial Informatics*.

[56] Hsu Y.-L., Yang S.-C., Chang H.-C., Lai H.-C. (2018). Human daily and sport activity recognition using a wearable inertial sensor network. *IEEE Access*.

[57] Buettner M., Prasad R., Philipose M., Wetherall D. Recognizing daily activities with RFID-based sensors.

[58] Hevesi P., Wille S., Pirkl G., Wehn N., Lukowicz P. Monitoring household activities and user location with a cheap, unobtrusive thermal sensor array.

[59] Rashidi P., Cook D. J. Mining Sensor Streams for Discovering Human Activity Patterns over Time.

[60] Shi S., Sigg S., Ji Y. Joint localization and activity recognition from ambient FM broadcast signals.

[61] Sekine M., Maeno K. (2012). Activity recognition using radio Doppler effect for human monitoring service. *Journal of Information Processing*.

[62] Jiang D., Li M., Xu C. (2020). WiGAN: a WiFi based gesture recognition system with GANs. *Sensors*.

[63] Kellogg B., Talla V., Gollakota S. Bringing gesture recognition to all devices.

[64] Qi X., Zhou G., Li Y., Peng G. Radiosense: exploiting wireless communication patterns for body sensor network activity recognition.

[65] Wang W., Liu A. X., Shahzad M., Ling K., Lu S. (2017). Device-free human activity recognition using commercial WiFi devices. *IEEE Journal on Selected Areas in Communications*.

[66] Wang F., Gong W., Liu J., Wu K. (2020). Channel selective activity recognition with WiFi: a deep learning approach exploring wideband information. *IEEE Transactions on Network Science and Engineering*.

[67] Wang F., Gong W., Liu J. (2019). On spatial diversity in WiFi-based human activity recognition: a deep learning-based approach. *IEEE Internet of Things Journal*.

[68] Wang S., Zhou G. (2015). A review on radio based activity recognition. *Digital Communications and Networks*.

[69] Zhang S., Wei Z., Nie J., Huang L., Wang S., Li Z. (2017). A review on human activity recognition using vision-based method. *Journal of healthcare engineering*.

[70] Ke S.-R., Thuc H. L. U., Lee Y.-J., Hwang J.-N., Yoo J.-H., Choi K.-H. (2013). A review on video-based human activity recognition. *Computers*.

[71] Jalal A., Uddin M. Z., Kim T.-S. (2012). Depth video-based human activity recognition system using translation and scaling invariant features for life logging at smart home. *IEEE Transactions on Consumer Electronics*.

[72] Ni B., Pei Y., Moulin P., Yan S. (2013). Multilevel depth and image fusion for human activity detection. *IEEE Transactions on Cybernetics*.

[73] Zhang H., Parker L. E. (2016). Code4d: color-depth local spatio-temporal features for human activity recognition from rgb-d videos. *IEEE Transactions on Circuits and Systems for Video Technology*.

[74] Gaglio S., Re G. L., Morana M. (2015). Human activity recognition process using 3-D posture data. *IEEE Transactions on Human-Machine Systems*.

[75] Yang X., Tian Y. (2017). Super normal vector for human activity recognition with depth cameras. *IEEE Transactions on Pattern Analysis and Machine Intelligence*.

[76] Ajmal M., Ahmad F., Naseer M., Jamjoom M. (2019). Recognizing human activities from video using weakly supervised contextual features. *IEEE Access*.

[77] Ayhan B., Kwan C., Budavari B., Larkin J., Gribben D., Li B. (2020). Video activity recognition with varying rhythms. *IEEE Access*.

[78] Raj R., Kos A. Different techniques for human activity recognition.

[79] Aggarwal J. K., Ryoo M. S. (2011). Human activity analysis: a review. *ACM Computing Surveys*.

[80] Enzweiler M., Gavrila D. M. (2009). Monocular pedestrian detection: survey and experiments. *IEEE Transactions on Pattern Analysis and Machine Intelligence*.

[81] Aggarwal J. K., Park S. Human motion: modeling and recognition of actions and interactions.

[82] Valera M., Velastin S. A. (2005). Intelligent distributed surveillance systems: a review. *IEE Proceedings - Vision, Image and Signal Processing*.

[83] Moeslund T. B., Hilton A., Krüger V. (2006). A survey of advances in vision-based human motion capture and analysis. *Computer Vision and Image Understanding*.

[84] Krüger V., Kragic D., Ude A., Geib C. (2007). The meaning of action: a review on action recognition and mapping. *Advanced Robotics*.

[85] Turaga P., Chellappa R., Subrahmanian V. S., Udrea O. (2008). Machine recognition of human activities: a survey. *IEEE Transactions on Circuits and Systems for Video Technology*.

[86] Candamo J., Shreve M., Goldgof D. B., Sapper D. B., Kasturi R. (2010). Understanding transit scenes: a survey on human behavior-recognition algorithms. *IEEE Transactions on Intelligent Transportation Systems*.

[87] Liu Y., Yuan J., Tu Z. (2022). Motion-driven Visual Tempo Learning for Video-Based Action Recognition. *IEEE Transactions on Image Processing*.

[88] Yurtsever M. M. E., Eken S. (2022). BabyPose: real-time decoding of baby’s non-verbal communication using 2D video-based pose estimation. *IEEE Sensors Journal*.

[89] Han C., Zhang L., Tang Y. (2022). Understanding and improving channel attention for human activity recognition by temporal-aware and modality-aware embedding. *IEEE Transactions on Instrumentation and Measurement*.

[90] He K., Gkioxari G., Dollár P., Girshick R. Mask r-cnn.

[91] Lin T.-Y., Dollár P., Girshick R., He K., Hariharan B., Belongie S. Feature pyramid networks for object detection.

[92] Yin Z., Wang X., Li L. Optimization of human body attitude detection based on mask RCNN.

[93] Wang Y., Wu J., Li H. (2020). Human detection based on improved mask R-CNN. *Journal of Physics: Conference Series*.

[94] Li X., Cheng S. Pedestrian gender detection based on mask R-CNN.

[95] Rosebrock A. (2017). Deep learning for computer vision with Python: starter bundle. *PyImageSearch*.

[96] Chollet F. Xception: deep learning with depthwise separable convolutions.

[97] Sandler M., Howard A., Zhu M., Zhmoginov A., Chen L.-C. Mobilenetv2: inverted residuals and linear bottlenecks.

[98] Tian Y., Zhang J. (2020). Optimizing sensor deployment for multi-sensor-based HAR system with improved glowworm swarm optimization algorithm. *Sensors*.

[99] Poulose A., Han D. S. (2019). Hybrid indoor localization using IMU sensors and smartphone camera. *Sensors*.

[100] Van Hauwermeiren J., Van Nimmen K., Van den Broeck P., Vergauwen M. (2020). Vision-based methodology for characterizing the flow of a high-density crowd on footbridges: strategy and application. *Infrastructure*.

